# Institutional review boards in Saudi Arabia: the first survey-based report on their functions and operations

**DOI:** 10.1186/s12910-023-00928-7

**Published:** 2023-07-10

**Authors:** Areej AlFattani, Norah AlBedah, Asma AlShahrani, Ammar Alkawi, Amani AlMeharish, Yasmin Altwaijri, Abeer Omar, M. Zuheir AlKawi, Asim Khogeer

**Affiliations:** 1 Biostatistics, Epidemiology and Scientific computing Department, King Faisal Specialist Hospital and Research Center, Riyadh, Saudi Arabia; 2 Neuroscience center, King Faisal Specialist Hospital and Research Center, Riyadh, Saudi Arabia; 3 Office of Research Affairs, King Faisal Specialist Hospital and Research Center, Riyadh, Saudi Arabia; 4 Research ethics monitoring office, King Abdulaziz City for Science and Technology, Riyadh, Saudi Arabia; 5 Research Department, The Strategic Planning Administration, General Directorate of Health Affairs Of Makkah Region, Ministry of Health, Makkah, 24382 Saudi Arabia; 6 Medical Genetics Unit, Maternity & Children Hospital, Makkah Healthcare Cluster, Ministry of Health, Makkah, 24382 Saudi Arabia

**Keywords:** Institutional review board, Research ethics committee, Approval, Functions, Roles, Review, Protocol

## Abstract

**Background:**

Institutional review boards (IRBs) are formally designated to review, approve, and monitor biomedical research. They are responsible for ensuring that researchers comply with the ethical guidelines concerning human research participants. Given that IRBs might face different obstacles that cause delays in their processes or conflicts with investigators, this study aims to report the functions, roles, resources, and review process of IRBs in Saudi Arabia.

**Method:**

This was a cross-sectional self-reported survey conducted from March 2021 to March 2022. The survey was sent to 53 IRB chairpersons and the administration directors (or secretary) across the country through email after receiving verbal consent. The validated survey consisted of eight aspects: (a) organizational aspects, (b) membership and educational training, (c) submission arrangements and materials, (d) minutes, (e) review procedures, (f) communicating a decision, (g) continuing review, and (h) research ethics committee (REC) resources. A total of 200 points indicated optimal IRB functions.

**Results:**

Twenty-six IRBs across Saudi Arabia responded to the survey. Overall, the IRBs in this study scored a total of 150/200 of the points on the self-assessment tool. Relatively newer IRBs (established less than ten years ago) conducted meetings at least once in a month, had annual funding, had more balanced gender representation, tended to score higher than older IRBs. The organizational aspect score was the lowest among all items in the survey (14.3 score difference, p-value < 0.01). The average turnaround time for expedited research from proposal submission to final decision was 7 days, while it was 20.5 days for the full committee review.

**Conclusion:**

Saudi IRBs performed generally well. However, there is room for focused improvement with respect to extra resources and organizational issues that require closer evaluation and guidance from the regulatory bodies.

**Supplementary Information:**

The online version contains supplementary material available at 10.1186/s12910-023-00928-7.

## Introduction

Institutional review boards (IRBs), and local ethics committees (LEC), the latter also known as research ethics committees (RECs), are official bodies that work to safeguard research participants and ensure their rights, safety and well-being throughout research that involves human subjects [1, 2]. Another important role of IRBs is to ensure that approved research are scientifically sound yet protecting the dignity and confidentiality of the participants [3, 4]. These roles were described in the Declaration of Helsinki (2013) [5] and the WHO 2011 Guidelines “Standards and operational guidance for ethics review of health-related research with human participants” [6]. To achieve their objectives, IRBs are essential checkpoints for all researchers throughout the various steps of their projects from proposal review, approval, to monitoring, and risk evaluation [7, 8].

The requirements for IRB operations and practices are described in the international council of harmonization protocol (ICH) guidelines which identifies criteria of IRBs compliance with the regulations. However, specific methods and policies must be implemented and generated by each institution to achieve the goal of protecting the rights and welfare of human research participants [9, 10]. Under the global standard of ICH regulations, IRBs can approve a research project, require modifications to the research proposal in order to gain approval, reject the protocol, or terminate or suspend the research study that has already received approval [11].

Despite their vital role in human research, there are many obstacles hindering the functions of IRBs. In low- and middle-income countries (LMICs), existing barriers include inadequate training of members, understaffing, limited resources, lack of diversity, and lack of rigorous ethical guidelines [7, 12, 13]. These barriers might affect expected IRB functions and draw criticism from investigators. IRBs that deliver unreasonable and inconsistent decisions may impose excessive bureaucracy and increase pressure on the research practice [14, 15], for e.g. researchers have reported frustration with the length of time taken for approvals [7, 16].

The turnaround time is one of the important indicators for IRB effective functions. In the United States, a survey of 68 U.S hospital IRBs found that only 26% required evidence of human subject research training and the time from submission to approval on average was 45 days [17]. In the United Arab Emirates, the time taken for proposal review in most IRBs was 56 days on average [18]. A survey-based study was conducted among RECs in Arab countries to assess IRBs functions that 50% organized continuing education for their members, 64% were established during the last 5 years, and 91% had standard operating procedures [7].

In Saudi Arabia, the National Committee of Bio-Ethics (NCBE) was created by royal decree on August 8th, 2001 to develop and monitor compliance with biomedical research ethics [19]. The NCBE published the “Law of Ethics of Research on Living Creatures” in 2010 (third update in 2022), which required the registeration of local IRBs before embarking on any research activity [20]. To be accredited, the legislation required all members of local ethics committees to complete an official training course on ethics and regulations and to register in the NCBE [21–23]. The course is implemented by the NCBE and contains universally recognized ethical principles which follow the guidelines of good clinical practices (GCP) and the standard operating procedures (SOPs) of the aforementioned Saudi law. To the best of our knowledge, although IRBs have existed in Saudi Arabia since the 1980s, the operations and functions of the local institutions have not been studied extensively. The goal of this study is to report data-driven figures about the operations and functions of IRBs in Saudi Arabia and to determine potential areas of improvement in their services.

## Methods

### Design and tool

This was a cross sectional survey-based study, using a self-administered questionnaire. The questionnaire was titled “Self-assessment Tool for Research Ethics Committees”. It was developed by the Middle East Research Ethics Training Initiative (MERETI) a network that was established in 2005 to provide training in research ethics to individuals from Arab countries [24]. The questionnaire has been validated and translated into Arabic by the original authors [25]. Written permission from authors of the questionnaire was obtained. Eight categories of questions were included: (a) organizational aspects, (b) membership and educational training, (c) submission arrangements and materials, (d) minutes, (e) review procedures, (f) communicating a decision, (g) continuing review, and (h) REC resources. The study survey takes 20 min to complete. We used this questionnaire for three reasons. First, it identifies the most crucial choices made in the research process to estimate a national score of the self-assessment tool for research ethics committees in Saudi Arabia. Second, to determine potential areas of improvement through identifying low scores among the examined categories. Third, to assess possible relationships between the categories such as: (organizational aspects) with (REC resources and submission arrangements), (membership and educational training) with (communicating a decision). The balanced gender representation of IRB members was defined as ratio of female/male: 40/60 as per the original questionnaire.

#### Population and sample size

We targeted the chairs and/or directors of all IRBs in Saudi Arabia accredited by National Committee of Bioethics (NCBE). NCBE oversees, regulates and monitors the local IRBs in KSA to their compliance with Sharia and statutory rules when dealing with biological material. The director oversees IRB Administration, runs meetings, and approves Human Subjects Research protocols that includes more than minimal risk. In case the office director was not available, the equivalent person involved in the operations and administrative work like the executive secretary was included. The total number of IRBs in KSA as of March 2019 when the proposal was approved was 53 committees. We planned to survey a sample of IRBs (the sampling unit) in Saudi Arabia by taking 2 participants (the chair and the research office director) from each IRBs. However, one respondent per IRB was sufficient. Sample size calculation was conducted using Stata version 17. Results indicated the required sample size to achieve 80% power at a significance criterion of α = 0.05 was N = 8 for the one-sample t-test. Thus, the obtained sample size of N = 26 IRBs deemed sufficient to test the study hypothesis [26].

#### Procedure

IRBs were approached after their contact information was obtained from an NCBE representative. An interviewer conducted a phone call to each IRB to invite the chairperson to join the study. To motivate for higher response, the interviewer explained the study’s importance and objectives to the chairperson then asked his/her verbal consent. In case the chairperson wasn’t available to take the call, the secretary or the office director was informed about the study and an email was sent immediately after the phone call. The email included an invitation, study information’s, a consent statement and a link to an online survey designed by *REDCap* to be filled out by the respondent. The link would remain valid for 20 days and reminders were sent to the recipient on the 3rd ,5th ,7th, 9th, 15th and 20th day to ensure higher response rates. If there was no response after 20 days, another phone call was carried out to remind the IRBs staff to fill it out. In case the IRB chairperson refused to participate, we asked for a brief reason to help us study the non-respondent group.

#### Statistical analysis

The full score of the assessment tool for optimal IBR functions and operations is 200. Each category was given up to five points. Data were recorded ,collected and stored in REDCap during the study period and saved with the principal investigator’s private documents, it will be stored for at least 5 years. Means of the scores were calculated with standard deviation if the distribution was normal. Medians and quartiles were calculated when the distribution was not normal. One sample t-test was used to assess the statistical significance of the observed scores versus the optimal ones. Student t-test was carried out to assess the relationships of the scores with different demographic variables, with a significance level of 0.05. Stata version 17.0 was used for statistical analysis and data visualization.

## Results

**Fig. 1:**
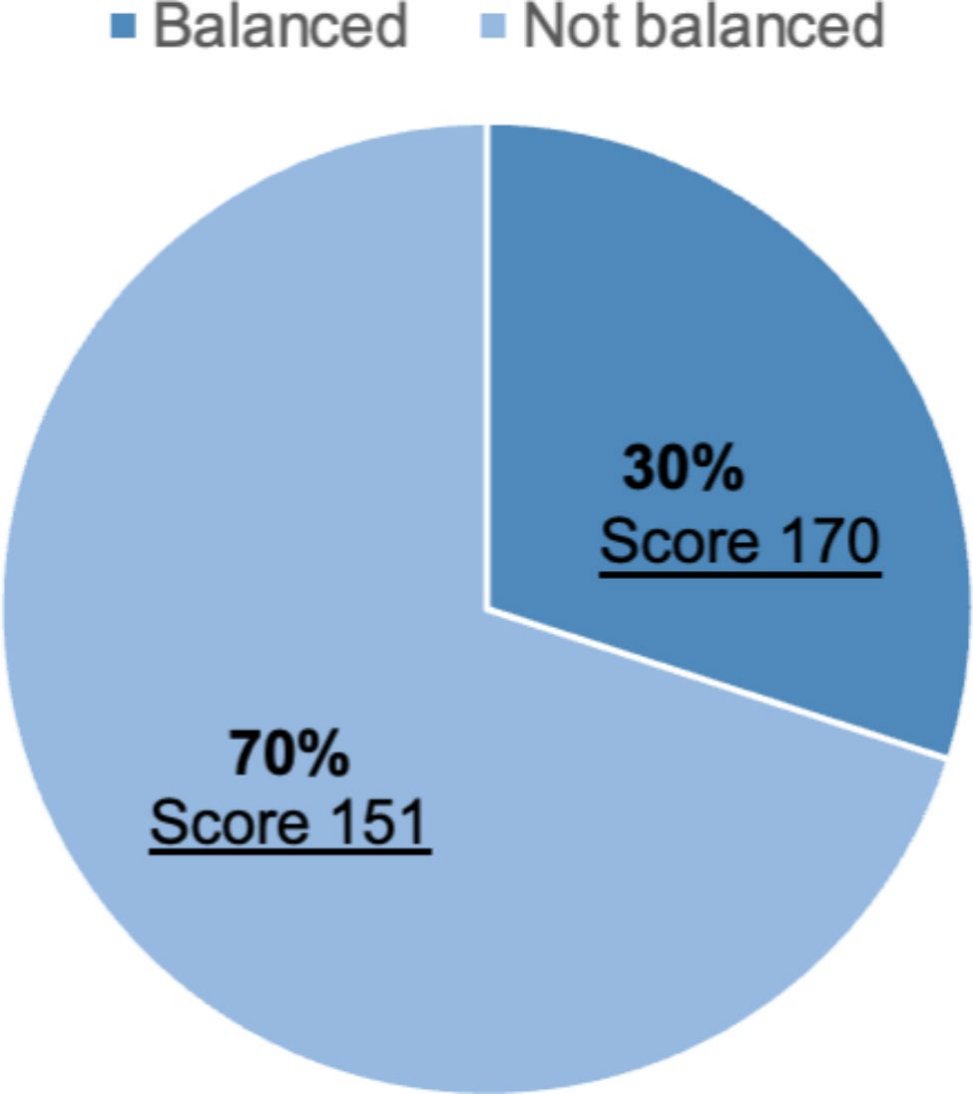
Balanced gender representation

We were able to collect data from 26 IRBs’ representatives (one per IRB: either the chair or the office director) across the KSA, with a 50% response rate. Around 70% of 24 IRBs had been running for less than 10 years with a mean total score of 152.23. The IRBs that had been running for 10 years and above had a mean total score of 140. Around a third of the IRBs used to have meetings less than once in a month whereas those who had meetings at least once a month comprised 68% of the sample. Most of the IRBs did not receive an annual budget and had a mean total score of 150, which is lower than that of IRBs who did receive an annual budget. Almost a third (N = 7) of the IRBs with balanced gender representation had a higher mean score of 170 compared to 16 IRBs with unbalanced gender representation, who had a mean score of 151. Table 1 shows the characteristics of the research ethics committees and the associated mean of total scores. IRBs that have been in existence for less than ten years conducted meetings at least once in a month, received an annual budget, and had balanced gender representation tend to score *higher* than other IRBs. The pie chart shows (Fig. 1 ) the proportion of balanced gender representation across the 26 IRBs. Undoubtedly, they were outnumbered by IRBs without balanced gender representation, who constituted 69.57% of the sample.

**Table 1:** Characteristics of research ethics committees and associated mean of total scores (N = 26)

Characteristic	Number (%)	Mean of the total score	*P values
Duration of existence < 10 years ≥ 10 years	17(70.83) 7(29.17)	152.23 140	0.87
Frequency of meeting At least once a month Less than once a month	17(68) 8(32)	153.47 160.5	0.50
Availability of annual budget Yes No	6(26.09) 17(73.91)	177.33 150.05	0.13
Balanced gender representation Yes No	7(30.43) 16(69.57)	170.42 151.37	0.07

* Mann–Whitney test

### Organizational aspects

A majority of IRBs (92.31%) had written standard operating procedures. The selection criteria for chair and IRBs members were: 92% prior research experience, 53.8% prior training in ethics, and 40% prior publications in ethics. Of the 26 IRBs, 18 (65%) indicated that they kept records electronically in a password-protected computer, while 7 (…?) of the IRBs filed records manually in a locked filing cabinet. Only one of the IRBs reported that they kept records on an open shelf as a way of storing and protecting the records; 76% and 80% of IRBs had a policy for disclosure and management of potential conflicts of interest for members and the research team, respectively. Only 40% had a quality Improvement program. Moreover, 60% reported that the organization evaluated the operations of the IRBs.

### Membership and educational training

The average number of IRB members was 10, with only 30.7% including a non-scientific member, and 73.08% requiring the investigators to undergo training in research ethics before submitting protocols for review. Approximately 70% of the IRBs conducted continuing education in research ethics for its members on a regular basis, and 84.62% documented the human subject protection training received by its members.

### Meeting Minutes

Of the 26 IRBs, 20 (77%) stated that meeting minutes reflected instances where members with a conflict of interest with respect to the protocols being discussed were excluded from the decision-making on the relevant protocols. Nineteen IRBs (73%) indicated that their minutes recorded the names of members who abstained from decision-making and the reason for abstention. Moreover, 22 (84.6%) affirmed that their minutes recorded the names of IRBs members who were excluded from the discussion and decision-making process due to a conflict of interest.

### Review of specific protocol items

Twenty-three respondents (88.46%) indicated that their IRBs evaluated whether the research protocol risks to research participants are reasonable in relation to the expected benefits to participants and society. However, only 9 (34.62%) indicated that their IRBs evaluated the methods used to protect the confidentiality of the research data collected.

### Total scores

The total score of each domain was compared to the optimal score. Interestingly, each domain had a highly significant p-value. The mean score for the organizational aspect was 39.691 ± 3.27, which indicated a 14-point gap from the optimal score (P < 0.001). Communicating a decision (approval letter) had an excellent mean score of 4.15 ± 1.59 and was only 1 point short of the optimal score, with P = 0.0120. Meanwhile, the total mean score was 150.15 ± 41.38 out of 200 points for all 26 IRBs (P < 0.001). Table 2 shows the mean score of each individual domain on the self-assessment tool.

**Table 2:** Scores of individual domains on the self-assessment tool (N = 26)

Domain	Mean$\pm$SD	Total possible points	*Score* *difference*	*P-value	*CI*
Organizational aspect	39.69$\pm$ 13.27	54	14.31	P < 0.001	34.33–45.05
Membership and educational training	21.23$\pm$8.89	30	8.77	P < 0.001	17.40- 24.51
Submission arrangement and materials	9.69$\pm$2.54	12	2.31	P < 0.001	8.66–10.71
Minuets	10.96$\pm$ 2.63	13	2.04	P < 0.001	9.89–12.02
Policies referring to review procedures	8.26$\pm$2.96	11	2.74	P < 0.001	7.07–9.46
Review of specific protocol items	34.53$\pm$9.73	43	8.47	P < 0.001	30.60- 38.46
Communicating a decision approval letter	4.15$\pm$ 1.59	5	0.85	P < 0.001	3.51–4.79
Continuing review	12.57$\pm$ 4.62	16	3.43	P < 0.001	10.70- 14.44
REC resources	9.30$\pm$3.39	16	6.7	P < 0.001	7.93–10.67
Total score	150.15$\pm$41.38	200	49.85	P < 0.001	133.49- 166.81

*One sample t-test

**Fig. 2:**
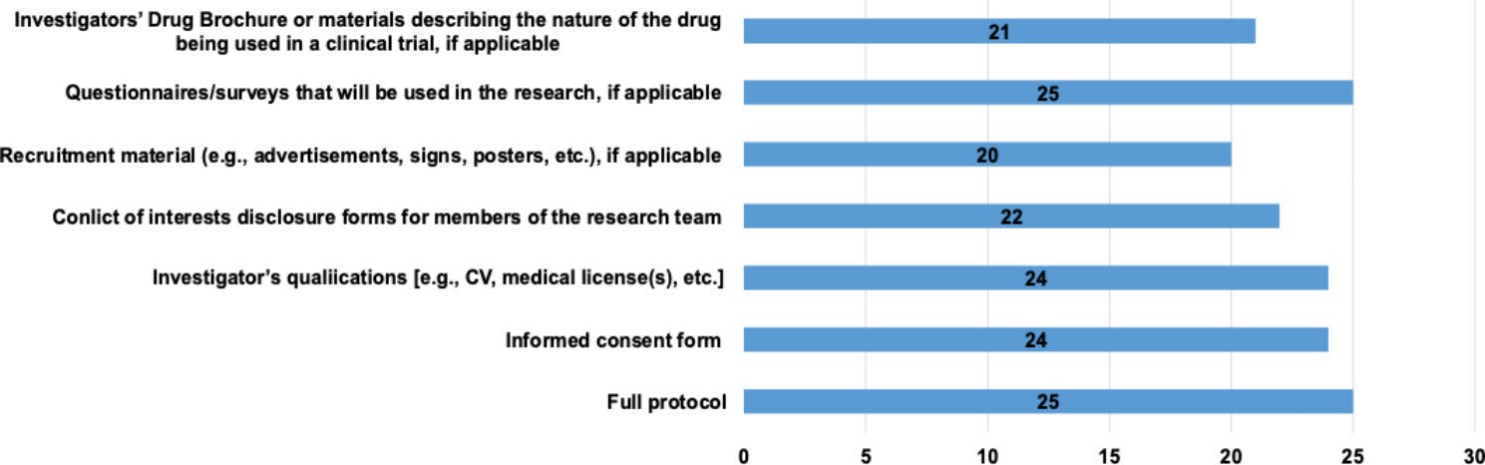
Submission materials are requested from the Principal Investigators when they submit their research protocol to the IRBs (N = 26)

The average meeting time was around an hour (67.5 min, ranging from 45 to 120) regardless of the number and type of protocols assessed. The average number of protocols reviewed by the IRBs in our sample was 40 annually. Most of these protocols were epidemiological or observational studies, while on average 4 protocols of clinical trials were reviewed annually. Meanwhile, the average number of protocols disapproved (i.e. rejected) and the average number of adverse events had the lowest median of 1 in a range of 0–30 and 0–5, respectively. As for our sample, the turnaround time for expedited research type from proposal submission to final decision was 7 days (range: 1–40 days) and 20.5 days (range: 3–70 days) for the full committee review. Table 3 shows the minimum, maximum, and median of the average IRBs workload. Figure 2 shows the submission materials which are requested from the Principal Investigators when they submit their research protocol to the IRBs.

**Table 3:** Workload parameters of the IRBs/RECs

Variable	Minimum	Maximum	Median
Average Duration of the meeting(minutes)	45	120	67.5
Average number of protocols reviewed annually	1	500	40
Average number of clinical trials reviewed annually*	0	50	4
Average number of epidemiologic/observational studies reviewed annually*	2	450	50
Average Number of new protocols reviewed by full committee**	1	60	8
Average Number of protocols disapproved***	0	30	1
Average Number of adverse reactions****	0	5	1
Average turnaround time from submission to approval of expedited research types in “Days”	1	40	7
Average turnaround time from submission to approval of Full committee research types in “Days”	3	70	20.5

*4 missing answer**7 missing answer ***6missing answer****11 missing answer

### Balanced gender representation

We assessed the association between each of the composite domains and balanced gender representation. IRBs with balanced gender representation tended to have higher scores on the composite domains compared to the IRBs with skewed gender balance. However, these differences were not statistically significant. It’s worth mentioning that three IRBs directors were female. Overall, the total mean score for all the composite domains with balanced gender representation was 169.42 ± 26.10 compared to IRBs with skewed gender balance, which had a total mean score of 151.37 ± 34.72, with P = 0.23 as shown in Table 4.

**Table 4:** The association between each of the composite domains and balanced gender representation

Domain	Balanced (n = 7) Mean$\pm SD$	Not balanced(n = 16) Mean$\pm SD$	*P-value*
Organizational aspect	$44.14\pm 10.82$	$39.81\pm 11.44$	0.40
Membership and educational training	$23.71\pm 9.25$	$21.06\pm 8.17$	0.49
Submission arrangement and materials	$10.71\pm 1.11$	$9.93\pm 1.84$	0.31
Minuets	$12.28\pm 1.49$	$11.18\pm 2.22$	0.24
Policies referring to review procedures	$9.71\pm 1.97$	$8.37\pm 2.5$	0.22
Review of specific protocol items	$39.14\pm 3.07$	$34.43\pm 8.30$	0.16
Communicating a decision approval letter	$4.85\pm 0.37$	$4\pm 1.65$	0.22
Continuing review	$14.85\pm 1.77$	$13\pm 4.11$	0.26
REC resources	$10\pm 4.04$	$9.5\pm 3.14$	0.75
Total score	$169.42\pm 26.10$	$151.37\pm 34.72$	0.23

## Discussion

This study is the first of its kind in KSA to assess the compliance of Saudi IRBs with international standards in terms of their structures and processes. The results identified areas where IRBs were performing well and where quality improvement was needed. The IRBs in this study scored a total of 150/200 of the points on the self-assessment tool.

The mean score for the organizational aspect was 39.69 ± 13.27, which was 14 points short of the optimal score. This low mean score can be explained by the low percentage of IRBs who included prior training and publications in ethics as the criteria for chair selection. Communicating a decision (approval letter) had an excellent mean score of 4.15 ± 1.59, which was only 1 point short of the optimal score. A case study of two medical college IRBs was conducted in Western India utilizing the Research Ethics Committee Quality Assurance Self-Assessment Tool (RECQASAT) -exactly same tool as ours [24] who scored 29 and 36 points for the organizational aspect, respectively. They both scored 3 points in the domain of communicating a decision. Meanwhile, the total mean score was 123 and 133 out of 200 for each REC individually, which is less than the total mean score concluded in this study [27]; this could indicate that IRBs in Saudi Arabia are performing better than some international IRBs.

According to the results obtained from a descriptive study conducted in Lebanon (2018) -using a modified version of the tool developed by Saleem et al. [24] where the total possible points was 175, the questionnaire mean score was 129.6 ± 22.3 out of 175, which was similar to the total possible score in the current study. This indicated an excellent adherence to international standards. Nevertheless, the organizational aspect was poorly scored, deviating by 39 points from the mean. Conversely, Lebanese RECs achieved the optimal total score for communicating a decision (approval letter) aspect [28].

A study conducted in Myanmar in 2020 showing the characteristics of the RECs and associated mean scores using the same tool by Saleem et al. [24, 29] found that that most of the 15 IRBs (93.3%) reported having meetings less than once a month; however, none of the RECs received an annual board meeting budget. Half of the IRBs with balanced gender representation reported a mean total score of 114.8 ± 15.3. While in the our study, 70% of the 25 IRBs who “had been running for less than 10 years” reported a mean total score of 152.23, the RECs that had been running for 10 years and above reported a lower mean total score (score = 140). Around a third of the IRBs used to meet less than once a month. Twenty-five per cent of the IRBs received a yearly budget, and they had a mean total score of 177.33. Only a third of IRBs had balanced gender representation.

According to another study conducted in low and middle-income countries included 64 IRBs/RECs [7], a slight majority reported having meetings at least once a month, while less than 40% stated they received an annual budget. Their total mean score was 137.7 ± 40.7 and 158.0 ± 19.2, respectively.

Turnaround time is one of the important indicators in the self-assessment tool. Our current study shows that the average turnaround time (TAT) from submission to approval for expedited research for committees that review up to 40 protocols annually is 7 days, and 20.5 days for the full committee research. However, the benchmark turnaround time according to the Association for the Accreditation of Human Research Protection Programs (AAHRPP) [30] is 30.2 and 44.9 for expedited and full committee approval respectively, for an average of 39.8 protocols reviewed annually. This indicates the efficiency of the Saudi IRBs/RECs evaluating system. However, this could be due to the type of protocol being reviewed as well. For instance, Saudi IRBs tended to review a higher number of observational and cross-sectional studies and a lower number of clinical trials annually which might be a factor in lowering the overall turnaround time.

More than half of the studied IRBs lacked gender-balanced representation. Nonetheless, Table 4 clearly demonstrates a better performance by gender-balanced IRBs in all individual domains, especially in the organizational aspect and in review of specific protocol items. This finding further supports previous evidence that such lack of diversity withholds IRBs from making scientifically sound decisions in developing countries [24]. While this could be due to gender segregation in the workplace that was effective until 2005 in Saudi Arabia, gender-balanced representation within IRBs should still be further monitored and improved to achieve women’s empowerment goals for the Saudi Vision 2030 [15].

### Limitations

First, a potential limitation of the current study is the self-assessment tool, which might cause exaggeration or underestimation of the function of the IRBs. In fact, it was not possible to look at the quality of the reviews/actual performance of IRBs. Some questions were broad like the duration of the meetings which didn’t give us specific view about study type. The non-probability sampling technique can also lead to a lack of generalizability. These results should be further compared to reports from the monitoring office of the Saudi IRBs for validation [31]. Second, the low response rate was an issue due to the existing formalities and bureaucratic hindrance in some places [32]. We faced different challenges and delays trying to approach IRBs, and many of them declined to participate without giving a specific reason for it. Ironically, others refused to participate because they insisted on a PI from the invited institution to be involved in the study in order to take IRB approval. We believe that these obstacles hinder improvement efforts by the NCBE and discourages the collaborations between institutions.

To overcome the suboptimal gender representation in IRBs, the following challenges should be addressed: the lack of financial incentives to attend IRB meetings, the current male-dominant situation in academia, and the lack of appreciation about the value that females can add to the decision-making process.

Furthermore, the high frequency of meetings in the older IRBs may indicate internal issues and difficulties, especially in terms of organizational aspects [29]. Some of the main difficulties were the lack of a dedicated annual budget for IRBs and the lack of protected time for IRBs members to review proposals and attend meetings.

Improving IRBs functions and operations will have a crucial role in accelerating clinical research procedures in Saudi Arabia, and attracting more sponsors to initiate research in local sites. Moreover, IRBs should be equipped to handle challenges of new research trends posing by the advance in knowledge, technology, and resources over the next decade [10]. Those challenges could be due to research type like: multicenter and multinational research, research with stored biological samples, artificial intelligence-related health research. On the other hand, challenges could arise from working with new entities involved in clinical research, such as contract research organizations, data and safety monitoring committees, clinical trial coordinating centers, and commercial IRBs [10, 33].

### Recommendations

Saudi IRBs need to be targeted for improvement with respect to balanced gender representation. Organizational issues might need closer investigation and guidance from the regulatory bodies. Moreover, training education and efficient support are required to maintain the key role of the institutions. These aspects need to be targeted for continuous improvement as well.

## Electronic supplementary material

Below is the link to the electronic supplementary material.

Additional File 1: Expanded table for values of most questions of the survey

## Data Availability

Data are available upon reasonable request from corresponding author email: aralfattani@kfshrc.edu.sa.
