# Supplementary material for: Institutional review boards in Saudi Arabia: the first survey-based report on their functions and operations
Source: BMC Med Ethics. 2023 Jul 10;24:50. doi: 10.1186/s12910-023-00928-7 (PMC10334562; doi:10.1186/s12910-023-00928-7)
Supplement: Supplementary file 1 — Additional File 1: Expanded table for values of most questions of the survey [file 12910_2023_928_MOESM1_ESM.pdf]

Question	%yes
4-Does the REC have written Standard Operating Procedures?	24(92.31)
6-Which of the following criteria are used to select the Chair of the REC? (Check all that apply.)<br>-prior training in ethics<br>-publication in ethics<br>-prior research experience<br>-Other	 <br>14(53.8)<br>10(38.4)<br>24(92.3)<br>2(7.6)
8-Which of the following criteria are used to select REC members? (Check all that apply.)<br>-prior training in ethics<br>-publication in ethics<br>-prior research experience<br>-Other	 <br>14(53.8)<br>12(46.1)<br>24(92.3)<br>3(11.5)
11-Does the REC have a quality improvement (QI) program for itself?	10(38.46)
13-Does the REC have a mechanism whereby enrolled research participants can file complaints or direct questions regarding human subject protection issues?	22(84.62)
14-How are records of REC stored?<br>-Paper folders in a locked file cabinet<br>-Electronic in a password protected computer<br>-On an open shelf<br>-Other	 6(23)<br>17(65.3)<br>1(3.8)<br>2(7.6)
2.5-Is there a requirement that the REC Chair (or the designee who is in charge of running the committee) has any prior formal training in research ethics?	15(57.69)
If yes, what type of training is required? (Check all that apply.)<br>-web based training<br>-workshop in research ethics<br>-course<br>-other	 13(50)<br>10(38.4)<br>7(26.9)<br>0(0)
2.6-Does the institution require that REC members have training in research ethics in order to be a member of the REC?	19(73.08)
If yes, what type of training is required? (Check all that apply.)<br>-web based training<br>-workshop in research ethics<br>-course<br>-other	 16(61.5)<br>8(30.7)<br>8(30.7)<br>0(0)
2.8-Does the REC conduct continuing education in research ethics for its members on a regular basis?	18(69.23)
3.1-Does the REC publish guidelines for submission of applications for the review by the REC?	24(92.31)
3.2-Does the REC require investigators to use a specific application form for the submission of their protocols to the REC?	8(30.77)
4.1-Do the minutes reflect that member were asked whether they had a conflict of interest regarding any of the protocols	20(76.92)

to be discussed and indicate that such members did not participate in the decision-making process of the relevant protocols?	
4.2-Do the minutes document that a quorum was present for all actions requiring a decision?	23(88.46)
4.3-Do the minutes document that all actions included at least one scientist in the review and participated in the decision-making process?	20(76.92)
4.4-Do the minutes document that all actions included at least one non-scientist in the review who participated in the decision-making process?	15(57.69)
4.5-Do the minutes document that all actions included at least one person who is not affiliated with the institution in the review and participated in the decision-making process?	17(65.38)
4.6- Do the minutes record the name of REC members who abstained from the decision-making process and provided the reason for abstention?	19(73.08)
4.7- Do the minutes record the name of REC members who were excused from the discussion and decision-making process due to a conflict of interest?	22(84.62)
4.8- Do the minutes reflect, when applicable, a discussion of the controversial aspects of the research protocol?	24(92.31)
5.3- Do REC members receive the protocol and other materials at a speciied time prior to the meeting?	23(88.46)
5.4- Does the REC require that reviewers use a checklist to document their ethical assessment of the research submission?	22(84.62)
5.8- Does the REC have a policy for how decisions are made (e.g., consensus or a vote)?	22(84.62)
5.9-Are members asked at the beginning of the meeting as to whether they had a conflict of interest regarding any of the protocols to be discussed and indicate that such members did not participate in the decision on the relevant protocols?	19(73.08)
5.11- Does the REC have a policy for follow-up review?	21(80.77)
6.1.3- Does the REC take into account prior scientific reviews, or do they review the appropriateness of the study design in relation to the objectives of the study, the statistical methodology, and the potential for addressing the objectives with the smallest number of research participants?	23(88.46)
6.2.1-Does the REC identify the different risks of the research protocol?	23(88.46)
6.2.5-Does the REC evaluate the importance of the knowledge to society that may reasonably be expected to result from the research?	22(84.62)
6.2.6-Does the REC evaluate whether the risks to research participants are reasonable in relation to any anticipated benefits to participants and the importance of the knowledge to be gained by society?	23(88.46)
6.4.1-Does the REC preserve privacy by evaluating the setting in which participants are recruited?	22(84.62)
6.4.2-Does the REC evaluate the methods for protecting the confidentiality of the collected research data?	9(34.62)
6.5.1-Does the REC review whether the potential benefits of the research are relevant to the health needs of the local community/country?	24(92.31)
6.5.2-Does the REC review whether any successful study product will be reasonably available to the concerned communities after the research?	20(76.92)

6.5.3-Does the REC review whether the community was consulted regarding the design and implementation of the research, if applicable?	16(61.54)

Research Ethics Committee (REC) Self -assessment Toolof Functions and Operations

In collaboration with the National Committee of Bioethics at KACST, We are pleased to invite you to participate in this survey. This research project is approved by IRB of King Faisal Specialist hospital and Research Centre with number RAC# 2191208. Thank you!

Are you willing to participate in this study?
- ◯ Yes
- ◯ No

Name of the IRB

____

A-ORGANIZATIONAL ASPECTS

What year was the REC established?

____

1. Is the REC subject to registration with a national authority? 2points
- ◯ Yes
- ◯ No

2. How often does the REC meet as a full committee to review research studies?
For meeting frequency equal or greater than once/month , 1point
- ◯ once/week
- ◯ once/month
- ◯ twice/month
- ◯ every two months
- ◯ other
- ◯ has not yet met to review protocol

specify others

____

3. Was the REC established under a high ranking authority (e.g., President's office, Ministry of Health, etc.)? 5 points
- ◯ Yes
- ◯ No

4. Does the REC have written Standard Operating Procedures? 5 points
- ◯ Yes
- ◯ No

5. Does the REC have a policy that outlines the process for appointing the REC Chair? 2 points
- ◯ Yes
- ◯ No

6. Which of the following criteria are used to select the Chair of the REC? (Check all that apply.) 1point each
- ☐ prior training in ethics
- ☐ publication in ethics
- ☐ prior research experience
- ☐ other

please describe others

____

7. Does the REC have a policy that describes the process for appointing the members of the REC and details the membership requirements and the terms of appointment? 2 point
- ◯ Yes
- ◯ No

8. Which of the following criteria are used to select REC members? (Check all that apply.) 1point each	☐ prior training in ethics<br>☐ publication in ethics<br>☐ prior research experience<br>☐ other (please describe)
Please describe others	__________________________
9. Does the REC have a policy for disclosure and management of potential conflicts of interest for the members of the REC? 5 points	◯ Yes<br>◯ No
10. Does the REC have a policy for disclosure and management of potential conflicts of interest for members of the research team? 5 points	◯ Yes<br>◯ No
11. Does the REC have a quality improvement (QI) program for itself? 5 points	◯ Yes<br>◯ No
If yes, describe what was done in the last year and any changes that were made as a result of the QI program.	________________________________
12. Does the institution/organization regularly evaluate the operations of the REC (e.g., budgetary needs, adequacy of material resources, adequacy of policies and procedures and practices, appropriateness of the membership given the research being reviewed, and documentation of the training requirements of the REC members)? 5 points	◯ Yes<br>◯ No
13. Does the REC have a mechanism whereby enrolled research participants can file complaints or direct questions regarding human subjects protection issues? 5 points	◯ Yes<br>◯ No
If yes, please describe the mechanism.	________________________________
14. How are records of the REC stored? 1 point each	◯ Paper folders in a locked file cabinet<br>◯ Electronic in a password-protected computer<br>◯ On an open shelf<br>◯ Other
Please describe others	__________________________
15. Does the REC require that there be a certain number of members present in order to make the meeting official to review protocols? 5 points	◯ Yes<br>◯ No

B- MEMBERSHIP AND EDUCATIONAL TRAINING

1. How many members are there on the REC? if ≥5 members 2 points

____

2.a How many are women?

____

2.b How many are men?
If F/M ratio between 0.4/0.6 then 2 points

____

3. Are any of the members not affiliated with the institution, that is, the member is not employed by the institution and is not related to a person who is employed? 2 points

◯ Yes
◯ No

4. Are any of the members considered to be a non-scientist? (A Non-Scientific member is any member who does not have a terminal degree in a medical or scientific field.) 2 points

◯ Yes
◯ No

Please note that one member may fulfill both criteria of non-scientist and non-affiliated, in which case, please check Yes for both #3 and #4.

5. Is there a requirement that the REC Chair (or the designee who is in charge of running the committee) has any prior formal training in research ethics? 5 points

◯ Yes
◯ No

If yes, what type of training is required? (Check all that apply.)

☐ web-based training
☐ workshop in research ethics
☐ course
☐ other (please describe)

Please describe others

____

6. Does the institution require that REC members have training in research ethics in order to be a member of the REC? 5 points

◯ Yes
◯ No

If yes, what type of training is required? (Check all that apply.)

☐ web-based training
☐ workshop in research ethics
☐ course
☐ other (please describe)

Please describe others

____

7. Does the institution require that investigators have training in research ethics in order to submit protocols for review by the REC? 5 points

◯ Yes
◯ No

If yes, what type of training is required? (Check all that apply.)

☐ web-based training
☐ workshop in research ethics
☐ course
☐ lecture
☐ other (please describe)

8. Does the REC conduct continuing education in research ethics for its members on a regular basis? 5 points	◯ Yes<br>◯ No
9. Does the REC document the human subjects protection training received by its members? 2 points	◯ Yes<br>◯ No

SUBMISSION ARRANGEMENTS AND MATERIALS

C- Submission Arrangements of Research Protocols 1 point each

	Yes	No
c1.Does the REC publish guidelines for submission of applications for the review by the REC?	◯	◯
c2.Does the REC require investigators to use a specific application form for the submission of their protocols to the REC?	◯	◯
c3.Does the REC have an informed consent template to help guide investigators in the writing of their informed consent forms?	◯	◯
c4.Does the REC require approval and signature of the department chair (or another individual) of the research protocol prior to the submission?	◯	◯
c5.Does the REC require a deadline for investigators to submit protocols for full committee review?	◯	◯

D- Submission Materials

Which of the following items are requested from the Principal Investigators when they submit their research protocol to the REC? 1 point each

	Yes	No
d1.Full protocol	◯	◯
d2.Informed consent form	◯	◯

d3.Investigator's qualifications [e.g., CV, medical license(s), etc.]	◯	◯
d4.Conflict of interests disclosure forms for members of the research team	◯	◯
d5.Recruitment material (e.g., advertisements, signs, posters, etc.), if applicable	◯	◯
d6.Questionnaires/surveys that will be used in the research, if	◯	◯
d7.Investigators' Drug Brochure or materials describing the nature of the drug being used in a clinical trial, if applicable	◯	◯

E- MINUTES

Does the REC maintain minutes of each meeting? 5 points

◯ Yes
◯ No

F- If minutes are kept, please answer the following questions regarding the minutes 1 point each

	Yes	No
f1.Do the minutes reflect that members were asked whether they had a conflict of interest regarding any of the protocols to be discussed and indicate that such members did not participate in the decision making process of the relevant protocols?	◯	◯
f2.Do the minutes document that a quorum was present for all actions requiring a decision?	◯	◯
f3.Do the minutes document that all actions included at least one scientist in the review and participated in the decision making process?	◯	◯
f4.Do the minutes document that all actions included at least one non-scientist in the review who participated in the decision making process?	◯	◯

	Yes	No
f5.Do the minutes document that all actions included at least one person who is not affiliated with the institution in the review and participated in the decision making process?	○	○
f6.Do the minutes record the name of REC members who abstained from the decision making process and provided the reason for abstention?	○	○
f7.Do the minutes record the name of REC members who were excused from the discussion and decision making process due to a conflict of interest?	○	○
f8.Do the minutes reflect, when applicable, a discussion of the controversial aspects of the research protocol?	○	○

G-POLICIES REFERRING TO REVIEW PROCEDURES 1 point each

	Yes	No
g1.Does the REC have a policy regarding how protocols will be reviewed?	○	○
g2.Does the REC bring in a consultant when necessary to provide scientific or other relevant expertise for review of a particular protocol?	○	○
g3.Do REC members receive the protocol and other materials at a specified time prior to the meeting?	○	○
g4.Does the REC require that reviewers use a checklist to document their ethical assessment of the research submission?	○	○
g5.Does the REC have a policy on the conditions for expedited REC review?	○	○

g6.Does the REC have a policy on the conditions for when studies may qualify for exempt status?	○	○
g7.Does the REC determine the interval of continuing review based on the risk of the study?	○	○
g8.Does the REC have a policy for how decisions are made (e.g., consensus or a vote)?	○	○
g9.Are members asked at the beginning interest regarding any the meeting as to whether they had a conflict of the protocols to be discussed and indicate that such members did not participate in the decision on the relevant protocols?	○	○
g10.Does the REC have a policy for communicating a decision?	○	○
g11.Does the REC have a policy for follow-up review?	○	○

H- REVIEW OF SPECIFIC PROTOCOL ITEMS 1 point each

	Yes	No
h1.Does the REC review the suitability of the investigators' qualifications to conduct the study?	○	○
h2.Does the REC review the adequacy of the clinical site, including the supporting staff, available facilities, and emergency procedures?	○	○
h3.Does the REC take into account prior scientific reviews or do they review the appropriateness of the study design in relation to the objectives of the study, the statistical methodology, and the potential for addressing the objectives with the smallest number of research participants?	○	○

I- Considerations of Risks and Benefits 1 point each

	Yes	No
i1.Does the REC identify the different risks of the research protocol?	◯	◯
i2.Does the REC determine whether risks have been minimized?	◯	◯
i3.Does the REC determine whether the risks are greater than minimal risk based on a written definition of minimal risk?	◯	◯
i4.Does the REC evaluate the probable benefits of the research to the participants?	◯	◯
i5.Does the REC evaluate the importance of the knowledge to society that may reasonably be expected to result from the research?	◯	◯
i6.Does the REC evaluate whether the risks to research participants are reasonable in relation to any anticipated benefits to participants and the importance of the knowledge to be gained by society?	◯	◯

J- Selection of Research Participants 1 point each

	Yes	No
j1.Does the REC review the methods to identify and recruit potential participants?	◯	◯
j2.Does the REC review recruitment processes to ensure that the selection of subjects will be equitable in regards to gender, religion, and ethnicity?	◯	◯

j3.Does the REC identify the potential of the research for enrolling participants who are likely to be vulnerable to coercion or undue influence (such as children, prisoners, persons with mental disabilities, or persons who are economically or educationally disadvantaged)?	◯	◯
j4.Does the REC consider the justification for including vulnerable populations in the research?	◯	◯
j5.Does the REC consider and require that additional safeguards be included in the study to protect the rights and welfare of the subjects?	◯	◯
j6.Does the REC consider the appropriateness of any financial or material incentives offered to participants for their participation in the research?	◯	◯

K- Privacy and Confidentiality	**1** point each	
	Yes	No
k1.Does the REC preserve privacy by evaluating the setting in which participants are recruited?	◯	◯
k2.Does the REC evaluate the methods for protecting the confidentiality of the collected research data?	◯	◯

L- Community Consultation	**1** point each	
	Yes	No
l1.Does the REC review whether the potential benefits of the research are relevant to the health needs of the local community/country?	◯	◯

l2.Does the REC review whether any successful study product will be reasonably available to the concerned communities after the research?	◯	◯
l3.Does the REC review whether the community was consulted regarding the design and implementation of the research, if applicable?	◯	◯

M- Safety Monitoring and Adequacy of Insurance to Cover Research-Related Injury 1 point each

	Yes	No
m1.Does the REC require, when appropriate, that the research plan include adequate provisions for monitoring the data collected to ensure the safety of subjects?	◯	◯
m2.Does the REC consider whether the sponsors of the research have adequate insurance to cover the treatments of injury related to the research?	◯	◯

N- Pediatric Research 1 point each

	Yes	No
Does the REC evaluate the need to obtain the child's assent?	◯	◯

O- Informed Consent 1 point each

	Yes	No
o1.Does the REC review the process by which informed consent will be obtained (e.g., how do investigators identify potential subjects, where does the informed consent process take place, are potential subjects allowed to take the consent form home and given enough time to ask questions, etc.)?	◯	◯

o2.Does the REC review which members of the research team will approach potential participants for their informed consent?	○	○
o3.Does the REC ensure that the informed consent document is understandable to the subject population?	○	○
o4.Does the REC waive the requirement to obtain informed consent that is based on written criteria?	○	○
o5.Does the REC waive the requirement to have a written signature on the informed consent document that is based on written criteria?	○	○

Suggested ways to assess the consent form might include:

- ☐ evaluate the reading level of the consent document
- ☐ have a community member read the consent form
- ☐ require investigators to assess subjects' understanding of the consent form

(Check all that apply)

P- Basic Elements of Informed Consent

Does the REC evaluate whether informed consent forms contain the following basic elements of informed consent? 1 point each

	Yes	No
p1.A statement that the study involves research	○	○
p2.An explanation of the purposes of the research	○	○
p3.The expected duration of the subject's participation	○	○
p4.A description of the procedures to be followed	○	○
p5.Identification of any experimental procedures	○	○
p6.A description of any reasonably foreseeable risks or discomforts to the participant	○	○

p7.A description of any benefits to the participant or to others that might reasonably be expected from the research	◯	◯
p8.A disclosure of appropriate alternative procedures or courses of treatment, if any, that might be advantageous to the subject	◯	◯
p9.A statement describing the extent, if any, to which confidentiality of records identifying the participant will be maintained	◯	◯
p10.For research involving more than minimal risk, an explanation as to whether any medical treatments are available if injury occurs and, if so, what the treatments consist of or where further information may be obtained	◯	◯
p11.An explanation of whom to contact for answers to pertinent questions about research	◯	◯
p12.An explanation of whom to contact for answers to pertinent questions about research participants' rights	◯	◯
p13.A statement that participation is voluntary	◯	◯
p14.A statement that refusal to participate will involve no penalty or loss of benefits to which the subject is otherwise entitled	◯	◯
p15.A statement that participant may discontinue participation at any time without penalty or loss of benefits to which the participant is otherwise entitled	◯	◯

Q- COMMUNICATING A DECISION

Please answer the following questions regarding the approval letter sent to the PI. If no approval letter is sent to the investigator, please skip this section 1 point each

	Yes	No
q1.Provide an expiration date that is 1 year from the date of the convened REC meeting in which the study was approved.	◯	◯
q2.Require the investigators to submit to the REC as an amendment any changes that occur in the research plan; for example, change in investigators, change in drug doses, change in the sample size, etc.	◯	◯
q3.Require the investigators to promptly report to the REC any adverse events or unanticipated problems.	◯	◯
q4.Require the investigators to promptly report to the REC any protocol deviations.	◯	◯
q5.Require investigators to use the REC-approved informed consent form that is stamped with an expiration date.	◯	◯

q6.CONTINUING REVIEW

R- Does the REC request a continuing review report from the investigators on at least a yearly basis?

◯ Yes
◯ No

R- If yes, which of the following items are requested in the continuing review report? 1 point each

	Yes	No
r1.Number of subjects enrolled	◯	◯
r2.Gender and ethnic/religious breakdown of enrolled subjects	◯	◯
r3.Number of subjects withdrawn from the research by the investigators	◯	◯
r4.The reasons for withdrawal	◯	◯

r5.Number of subjects who dropped out of the research	◯	◯
r6.The reasons why subjects dropped out	◯	◯
r7.Verification that informed consent was obtained from all subjects and that all signed consent forms are on file	◯	◯
r8.Number and description of serious adverse events in the previous year (SAEs)	◯	◯
r9.List of any protocol violations or deviations	◯	◯
r10.Any safety monitoring report	◯	◯
r11if the study is completed, submit a final report describing the study results.	◯	◯

S- Does the REC(s) have its own yearly budget? 5 points	◯ Yes<br>◯ No
s1.If yes, is there a budget for training of administrative staff and REC members? 1 point	◯ Yes<br>◯ No
s2.Please check below the physical resources of the REC (check all that apply): 1 point each	☐ access to a meeting room<br>☐ access to a computer and printer<br>☐ access to the internet<br>☐ access to a facsimile<br>☐ access to cabinets for storage of the protocol files<br>(Check all that apply)
T-Does the REC have administrative staff assigned to the REC? 5 points	◯ Yes<br>◯ No
t1.Is the person full-time?	◯ Yes<br>◯ No
t2.Is the person half-time?	◯ Yes<br>◯ No

U- WORKLOAD OF THE REC

u1.Average Duration of the meeting	__________________________
u2.Average number of protocols reviewed annually?	__________________________
u3.Average number of clinical trials reviewed annually?	__________________________

u4.Average number of epidemiologic/observational studies reviewed annually? ________________________________

After a brief review of three recent REC minutes, complete the following table with a specific number or N/A (not applicable).

u5.Average Number of new protocols reviewed by full committee ________________________________

u6.Average Number of protocols disapproved ________________________________

u7.Average Number of adverse reactions ________________________________

u8.Average Number of continuing review protocols approved by expedited review that were reported to the REC ________________________________

u9.Average Number of continuing review protocols reviewed by full committee ________________________________

u10.Average Number of amendments approved by expedited review that were reported to the REC ________________________________

u11.Average Number of amendments reviewed by full committee ________________________________

u12.Average turnaround time from submission to approval of expedited research types in "Days" ________________________________

u13.Average turnaround time from submission to approval of Full committee research types in "Days" ________________________________

V- Comments or suggestions: ________________________________
